# The Role of Mitochondrial Copy Number in Neurodegenerative Diseases: Present Insights and Future Directions

**DOI:** 10.3390/ijms25116062

**Published:** 2024-05-31

**Authors:** Annamaria Cerantonio, Luigi Citrigno, Beatrice Maria Greco, Selene De Benedittis, Giuseppe Passarino, Raffaele Maletta, Antonio Qualtieri, Alberto Montesanto, Patrizia Spadafora, Francesca Cavalcanti

**Affiliations:** 1 Institute for Biomedical Research and Innovation, National Research Council (IRIB-CNR), 87050 Mangone, CS, Italy; annamaria.cerantonio@irib.cnr.it (A.C.); patrizia.spadafora@irib.cnr.it (P.S.);; 2 Department of Biology, Ecology and Earth Sciences, University of Calabria, 87036 Rende, CS, Italy; 3 Regional Neurogenetic Centre (CRN), Department of Primary Care, ASP Catanzaro, 88046 Lamezia Terme, CZ, Italy; 4 Association for Neurogenetic Research (ARN), 88046 Lamezia Terme, CZ, Italy

**Keywords:** neurodegenerative diseases, mitochondrial function, mitochondrial DNA copy number

## Abstract

Neurodegenerative diseases are progressive disorders that affect the central nervous system (CNS) and represent the major cause of premature death in the elderly. One of the possible determinants of neurodegeneration is the change in mitochondrial function and content. Altered levels of mitochondrial DNA copy number (mtDNA-CN) in biological fluids have been reported during both the early stages and progression of the diseases. In patients affected by neurodegenerative diseases, changes in mtDNA-CN levels appear to correlate with mitochondrial dysfunction, cognitive decline, disease progression, and ultimately therapeutic interventions. In this review, we report the main results published up to April 2024, regarding the evaluation of mtDNA-CN levels in blood samples from patients affected by Alzheimer’s (AD), Parkinson’s (PD), and Huntington’s diseases (HD), amyotrophic lateral sclerosis (ALS), and multiple sclerosis (MS). The aim is to show a probable link between mtDNA-CN changes and neurodegenerative disorders. Understanding the causes underlying this association could provide useful information on the molecular mechanisms involved in neurodegeneration and offer the development of new diagnostic approaches and therapeutic interventions.

## 1. Introduction

### 1.1. Common Aspects of Neurodegenerative Diseases

Neurodegenerative diseases are conditions affecting the central nervous system (CNS) that progressively lead to neuronal damage and represent a leading cause of death among the elderly worldwide. The molecular mechanisms that lead to the onset of neurodegenerative diseases are not yet fully understood. Abnormal protein accumulation, cellular damage exacerbated by oxidative stress, loss of neuromuscular control and changes in mitochondrial function are considered the potential causative promoters [1].

Alzheimer’s disease, Parkinson’s disease, Huntington’s disease, amyotrophic lateral sclerosis and multiple sclerosis are the most prevalent forms of neurological disorders [2]. Common pathological denominators are the accumulation of insoluble protein aggregates in neurons, glial cells and decreased sensory–motor and cognitive functions [3]. 

In particular, AD is characterized by the accumulation of phosphorylated microtubule-associated protein tau in neurofibrillary tangles and amyloid-β (Aβ) deposits; PD presents neuronal accumulation of α-synuclein in Lewy bodies dementia; HD is a monogenic progressive neurodegenerative disorder caused by the expansion of a Cytosine-Adenine-Guanine (CAG) repeat in the first exon of the Huntingtin gene (*HTT*), triggering the accumulation of toxic oligomers of the mutant huntingtin protein [4]; ALS is characterized by aggregates of TAR DNA-binding protein 43 (*TDP43*) [5,6,7]. Furthermore, MS is an autoimmune disease characterized by inflammatory demyelination, deterioration of axonal terminal structures, and subsequent neurological damage [8]. 

The increase in life expectancy in several countries resulted in an increase in the prevalence of these disorders, especially in the age group between 70 and 80 years, with PD and AD as the most common forms [9]. So far, there is no effective treatment for neurodegenerative diseases. Therapeutic interventions aim to reduce cognitive, sensory and motor symptoms to improve the survival of affected individuals [10]. 

### 1.2. Mitochondrial DNA Structure and Function

Mammals contain several identical copies of mtDNA, consisting of a 16,569 bp double-stranded circular molecule encoding 13 respiratory complex subunits, 2 ribosomal RNAs and 22 transfer RNAs. The encoded proteins are located in the inner mitochondrial membrane where they take part, as subunits, in the respiratory complexes I, III, IV and V of the oxidative phosphorylation (OXPHOS) system. A control region, called a displacement loop (D-loop), contains the heavy-strand origin of replication (OriH) where the production of new mtDNA molecules begins [11,12,13].

Mitochondrial transmission follows a maternal inheritance. The polyploid nature of the mitochondrial genome—up to several thousand copies per cell—gives rise to important features of mitochondrial genetics, known as homoplasmy and heteroplasmy. Homoplasmy occurs when all copies of the mitochondrial genome are identical, whereas heteroplasmy occurs when there is a mixture of two or more mitochondrial genotypes. The rate of mutated versus wild-type mtDNA molecules is the discriminant element between pathological and normal phenotypes in the spectrum of mitochondrial diseases [14].

### 1.3. Mitochondrial DNA Alterations as Key Players of Aging

The hallmarks of aging are the decline in mitochondrial function, morphology and the mtDNA content. The accumulation of somatic mtDNA alterations, deletions and point mutations in different tissue is known to induce aging phenotypes [15].

Furthermore, mitochondria preserve cellular homeostasis by producing reactive oxygen species (ROS) in several cellular compartments, maintaining both respiration capacity and efficiency [16,17]. 

It has been suggested that oxidative stress is another biological parameter affected by mitochondrial changes in aging. During aging, mitochondrial function deteriorates and the adaptive capacity of the cells to face oxidative stress decreases. This process contributes to exacerbating the negative relationship between stress and longevity, defined as a “toxic excitation effect” [18].

The progressive alteration of mitochondria results in increased production of ROS that, in turn, cause mitochondrial dysfunction, mitochondrial enlargement and injury, leading to a ribosome detachment in the rough endoplasmic reticulum (ER). The compromise of ER morphology results in DNA damage, shortening of telomeres, lipid peroxidation and oxidative modification of proteins, triggering cellular oxidative stress and senescence. Cellular senescence promotes the secretion of senescence-associated secretory phenotypes (SASPs), including IL-6, IL-8, and TNF-α, involved in the promotion of chronic inflammation and systemic aging [19].

Disorganization in mitochondrial structure reduces the mtDNA’s ability to repair cellular damage and also promotes the neuro-aging process. Aging compromises the functionality of the mitochondrial permeability transition pores (mPTPs), resulting in depolarization of mitochondria and OXPHOS uncoupling. ROS are able to stimulate mPTPs, the release of cytochrome-C oxidase and the initiation of the caspase-9 cascade, triggering the activation of the apoptotic pathway. mPTPs, under ROS activity, are also able to activate the nod-like receptor pyrin domain 3 (NLRP3), an important promoter inastrocyte inflammation, and culminate in motor impairments and cognitive abnormalities [20].

It has been speculated that the mitochondrial respiratory chain generates free radicals able to perturb cell molecules over time. This hypothesis gave rise to the theory that several molecules may accumulate longitudinal damage, showing a progressive functional decline, and are thus considered an aging “clock” [21].

Mitochondrial mitophagy, the system responsible for eliminating damaged mitochondria, undergoes functional decline during aging. The mtDNA replication rate is higher than that of nuclear DNA, but its capability to correct errors happening during this process is less efficient, contributing to the accumulation of mtDNA mutations. Furthermore, mtDNA polymerase activity is more likely to be damaged by ROS owing to its specific location, thus affecting mtDNA polymerase gamma fidelity and vulnerability [22].

In the past, the identification of mtDNA alterations was not easy due to their low abundance in cells or tissues and the resulting limitations of existing technologies. The advent of next-generation sequencing (NGS) helped researchers to deeply characterize mtDNA and its different types of variants during the aging process [23]. Several studies have quantified mtDNA copy number (mtDNA-CN) in tissues of individuals at different ages. Ding et al., 2015 reported an age-associated decrease in mtDNA levels measured in lymphocytes from over 2000 Sardinians [24]. 

Furthermore, in older people, a lower mtDNA-CN has been associated with a progressive cognitive and physical decline and with a higher mortality risk [25]. Moreover, the pathological A3243G mutation on mtDNA has been strongly correlated with reduced strength and cognitive and metabolic impairment in the elderly [26]. Finally, mutations in mtDNA have been reported in the D-loop region that is the most susceptible to genome instability. In particular, T414G transversion has been detected in up to 50% of mtDNA molecules of individuals above 65 years of age [27].

### 1.4. The Contribution of mtDNA and mtDNA Copy Number Content in the Onset of Neurodegenerative Diseases

Mitochondrial dysfunctions play a crucial role in the onset of neurodegenerative disorders, just as in aging. The amount of mtDNA-CN is usually linked to energy storage and production, oxidative stress trends and mitochondrial membrane potential [28]. 

A decrease in mitochondrial protein expression, alterations in cellular morphology and lower respiratory enzyme activity were observed in knockdown cell models of reduced mtDNA-CN, demonstrating the relationship between mtDNA-CN and mitochondrial function [29]. The evaluation of intracellular mtDNA-CN in immune cells from peripheral blood has been associated with mitochondrial diseases, cancer and cardiovascular diseases [30,31]. Although the pathogenesis of many neurodegenerative diseases has not yet been clarified, some evidence suggests that changes in mtDNA content could play a crucial role in the development of such disorders, for which establishing a timely diagnosis and predicting disease progression is still demanding [32]. Alteration in mtDNA levels, observed in multiple tissues of the body, could be the cause of progressive bioenergetics deficits and functional defects in the OXPHOS pathway, culminating in changes in the respiratory activity of neurons and in neurodegeneration [33].

It is known that neurodegenerative processes can start before the appearance of clinical manifestations, and alteration in mtDNA-CN may be the sign of initial cognitive decline [34]. All these findings support the contribution of mtDNA-CN as a candidate biomarker in the pathogenesis of neurodegeneration, even in the early stages of disease, because its alteration could reflect mitochondrial biogenesis and function.

The significance of mtDNA-CN variability in blood is widely investigated, but often the conclusions are discordant due to poor reproducibility of the results, small sample sizes, and differences existing between ethnic groups. The aim of our review is to provide the current outcomes achieved by evaluation of mtDNA-CN content in neurodegenerative diseases, highlighting the importance of using this parameter to better understand the onset and symptomatology of these disorders.

### 1.5. Relevant Sections

In this review, we reported all the published scientific papers supporting the hypothesis that evaluation of mtDNA-CN in biological fluids and tissues, like blood, could provide important indications about the onset and progression of neurodegenerative diseases. 

All the relevant clinical and molecular findings are summarized in Table 1, Table 2, Table 3, Table 4 and Table 5.

### 1.6. Alzheimer’s Disease

Rodriguez-Santiago et al. [35] investigated mtDNA-CN alterations in brain regions (cerebellum, hippocampus and frontal cortex) of 12 AD necropsies and in blood specimens of 17 Spanish AD patients versus 11 healthy controls. They performed quantitative real-time PCR (qPCR) using primers that detected the *mtND2* and *r18S* gene sequences. There were no differences in terms of mtDNA-CN in blood specimens, cerebellum and hippocampus brain regions of AD patients compared to healthy controls. Interestingly, a reduction in mtDNA content was observed in AD frontal cortex, which is the area where neuronal loss often occurs, suggesting that mtDNA alterations could have an anatomic specificity (Table 1). Delbarba et al. [36] evaluated mitochondrial enzymatic activity and mtDNA-CN from peripheral blood mononuclear cells (PBMCs) in a Spanish cohort of 20 AD patients, 24 mild cognitive impairment (MCI) individuals and 30 healthy controls. Moreover, DNA samples derived from an Italian cohort of 276 AD patients, 70 MCI patients and 248 healthy controls underwent mtDNA-CN analysis. mtDNA-CN was evaluated by qPCR using specific primers for the cytochrome B (*CYT B*) gene and normalized to the acidic ribosomal phosphoprotein P0 (*Arbp/36B4*) nuclear gene. *CYT B* levels were reduced in AD, while cytochrome C (*CYT C*) decreased in both AD and MCI subjects. Cytochrome C oxidase and citrate synthase activity were higher in MCI individuals compared to controls, while AD patients only showed increased CYT C oxidase activity. In both Spanish and Italian cohorts, mtDNA content was lower in AD and MCI patients compared to controls. However, the mean values of Spanish MCI mtDNA content were higher than Italian MCI DNA samples. Authors attributed this discrepancy to the different mini-mental state examination (MMSE) score, which was two points higher in Spanish MCI compared to Italian MCI patients. Spanish AD patients also showed a significant reduction in peroxisome proliferator-activated receptor gamma coactivator 1-alpha (PGC-1α) and mitochondrial transcription factor A (TFAM) levels compared to controls, while only TFAM levels were decreased in MCI Spanish patients. The study supported the fact that decreased mtDNA content, detected in early AD stage, might help in the differential diagnosis and could be potentially a blood-based signature of AD (Table 1). 

Lunnon et al. [37] measured nuclear- and mitochondrial-encoded OXPHOS genes and mtDNA content in peripheral blood samples in a cohort of English patients. qPCR was used to measure gene expression levels of 12 mitochondrial-genome-expressed transcripts in 164 AD patients, 168 MCI patients and 177 healthy controls. Luminex was used to quantify levels of functional ETC proteins in 24 AD patients, 19 MCI patients and 27 healthy controls. qPCR for mtDNA-CN analysis (conducted in 28 AD subjects, 31 MCI subjects and 28 healthy controls) was performed by a QuantiTect SYBRgreen PCR kit (Qiagen) using primers complementary to unique regions and genes of the mitochondrial genome. The single-copy nuclear gene *beta 2 microglobin* (*B2M*) was used to quantify mtDNA content as the ratio of mitochondrial genome to nuclear genome. The majority of nuclear-encoded OXPHOS genes had lower expression in MCI and AD patients compared to controls. On the other hand, mitochondrial-encoded OXPHOS genes had higher expression in MCI and AD compared to controls. However, no differences in mtDNA-CN between AD, MCI and healthy controls were found (Table 1). Liou et al. [38] determined the thiobarbituric acid reactive substances (TBARS), thiols and mtDNA-CN levels in a cohort of 600 Taiwanese AD subjects compared to 601 healthy controls. Patients were divided based on the Ɛ4 allele pattern of the *APOE* gene (no Ɛ4 allele, heterozygous Ɛ4 allele, homozygous Ɛ4 allele) that represents a significant risk factor for AD onset. mtDNA content was measured by qPCR using primers complementary to the sequence of the *ND1* gene and normalized to the *β-actin* gene. Results showed a higher TBARS and a lower thiols level in the AD cohort compared to healthy controls, while mtDNA-CN levels were lower in the AD group compared to controls. A significant trend of lower mtDNA-CN was correlated with the presence of the homozygous *APO Ɛ4* allele in the AD cohort. The authors also investigated mtDNA-CN changes in AD patients receiving cholinesterase inhibitors for more than 3 months versus AD patients with no history of medication. They found lower TBARS levels and higher mtDNA content in treated AD patients compared to the non-medication group (Table 1). 

Gorham et al. [39] investigated mtDNA-CN in blood with the purpose of identifying population-specific differences as indicators of mitochondrial stress and dysfunction associated with AD risk. They focused on the Mexican–American (MA) population, which is the fastest-aging population in the United States and therefore the most susceptible to AD. They evaluated mtDNA-CN in MA individuals (31 AD patients, 72 MCI subjects and 149 healthy controls) in comparison to non-Hispanic white (NHW) subjects (63 AD patients, 28 MCI subjects and 129 healthy controls). mtDNA content was measured by qPCR via the mitochondrial minor arc (MinArc) and the nuclear *B2M* gene. mtDNA-CN decreased in AD patients compared to controls in the MA cohort. The same trend was not observed in the NHW cohort, in which a significant mtDNA-CN increase was observed only in MCI subjects. The authors concluded that there was a significant relationship between mitochondrial dysfunction and cognitive decline in both cohorts (Table 1).

### 1.7. Parkinson’s Disease

Gui et al. [40] examined mutations in the D-loop region and mtDNA-CN in 414 Chinese PD patients and 231 healthy subjects. Mutations in the D-loop region were examined by direct sequencing of the 982-bp amplified PCR product. qPCR was performed using primers for specific amplification of the mtDNA D-loop region and the nuclear DNA-encoded *β-actin* gene as normalizer. mtDNA-CN decreased in PD patients compared to controls, and lower mtDNA-CN was more frequently detected in the oldest PD patients. Furthermore, mtDNA-CN in PD patients who had mutations at the poly-C stretch or close to the heavy-strand replication origin sites was significantly decreased compared to PD patients without D-loop mutations. The authors also investigated the role of DNA polymerase subunit gamma 1 (*POLG1*) mutations in the landscape or mtDNA content. In fact, mutations in *POLG1*, responsible for mtDNA replication and repair activity, result in dysfunction in the respiratory chain. mtDNA-CN in PD patients having mutations in the *POLG1* gene (R232H, W748S and intronic SNPs c2070-12T>A and c2070-64G>A) was lower than in the PD cohort without *POLG1* mutations. This study provided evidence that mtDNA content may be involved in PD and could have potential prognostic significance in PD progression (Table 2). Pyle et al. [41] studied mtDNA content in multiple tissues from English PD patients (363 peripheral blood samples, 151 Substantia nigra pars compacta [SNpc] samples and 120 frontal cortex [FC] tissue samples) and matched control subjects (262 peripheral blood samples, 33 SNpc samples and 37 FC tissue samples). Evaluation of mtDNA-CN was performed by multiplex Taqman qPCR amplification of the mitochondrial genes *MTND1* and *MTND4* and the nuclear-encoded gene *B2M*. A significant mtDNA-CN reduction was observed in peripheral white blood cells (PBCs) in PD patients compared to controls. Analysis of isolated brain tissues revealed a similar reduction in mtDNA-CN in SNpc of PD patients. Furthermore, authors identified a correlation between reduced mtDNA-CN in PBCs and smoking history in PD patients (Table 2).

Stoccoro et al. [42] investigated D-loop regulatory region methylation levels and mtDNA-CN in peripheral blood samples of 30 Italian PD patients and 30 healthy controls. DNA methylation was performed using methylation-sensitive high-resolution melting (MS-HRM) and pyrosequencing techniques. Quantification of mtDNA-CN was performed by qPCR using primers specific for a nuclear DNA region and mtDNA region. Authors did not find differences in D-loop methylation levels or in mtDNA-CN between PD and controls, probably due to the small sample size of both cohorts (Table 2). Müller-Nedebock et al. [43] evaluated mtDNA-CN alteration in blood of 72 African-ancestry PD patients compared to 79 healthy controls. A droplet digital polymerase chain reaction (ddPCR) was performed to quantify mtDNA-CN levels as a ratio of a mitochondrial gene (*MT-TL1*) to a nuclear gene (*B2M*).

Authors reported significantly higher mtDNA-CN levels in whole blood of PD cases compared to controls. Due to the differences in mtDNA-CN, the authors suggested that this result might be a consequence of unaccounted-for factors and variability in study designs (Table 2). Asghar et al. [44] investigated the association between telomere length (TL) and mtDNA-CN in blood of 100 Swedish PD patients and 12 healthy controls. Furthermore, they investigated the association between PD, Parkinson’s disease dementia (PDD), and dementia with Lewy bodies (DLB) with cellular aging biomarkers. TL and mtDNA-CN were measured by qPCR using the ScienceCell kit. Genomic human DNA of known TL and mtDNA-CN were used as reference for data analysis. Results showed that, in blood, PD patients had lower mtDNA-CN and higher TL compared to controls and that TL was positively correlated with levodopa equivalent daily dose (LEDD) in PD patients. Even in PD brain tissues, telomeres were longer compared to controls. On the contrary, mtDNA-CN was lower compared to controls, with the lowest values detected in PDD and DLB postmortem tissues (Table 2).

Ortega-Vazquez et al. [45] evaluated TL and mtDNA-CN in a Mexican cohort of 27 naive PD patients and 22 healthy controls. PD patients were also studied after one or two years of dopaminergic replacement therapy (DRT). qPCR for TL was performed by the amplification of telomeric sequence and a single-copy control gene (*b-globin*) as reference. mtDNA-CN was evaluated by qPCR using primers complementary to the *ND3* gene and nuclear tyrosine hydroxylase (*TH)* gene as reference. TL was significantly shorter in PD patients compared to controls, even after DRT administrations. In contrast, mtDNA-CN in untreated PD patients was similar to that of controls. However, this value increased after dopaminergic treatment (Table 2).

### 1.8. Huntington’s Disease

Chen et al. [46] examined 8-hydroxydeoxyguanosine (8-OHdG), plasma malondialdehyde (MDA), mtDNA-CN, mitochondrial enzyme Cu/Zn-superoxide dismutase (Cu/Zn-SOD), and glutathione peroxidase (GPx) activities in blood of 16 Taiwanese HD patients and 36 healthy controls.

High-performance liquid chromatography–electrochemical detector (HPLC-ECD) and HPLC techniques were used to measure 8-OHdG and MDA levels, respectively, while Cu/Zn-SOD activity was determined using a RANSOD kit (RANDOX, County Antrim, UK). A TaqMan PCR Core Reagent kit was used to quantify mtDNA-CN and deleted mtDNA molecules using primers specific for mitochondrial genes (*ND1*, *CYTB*, *COX1*) and nuclear genes (*GAPDH*). The 8-OHdG and MDA levels resulted significantly higher in HD patients than in controls, while GPx and Cu/Zn-SOD activity was reduced in HD patients compared to controls. Higher mtDNA-deleted and mtDNA-CN levels were found in HD patients compared to controls, probably due to a compensatory response to an increased oxidative stress and a reduction in antioxidant enzymes involved in the cell defense mechanisms (GPx and Cu/Zn-SOD) (Table 3). 

Liu et al. [47] investigated the role of mtDNA content in blood leukocytes as a biomarker to predict the severity of clinical manifestation in poly-Q diseases. They recruited 17 HD Taiwanese patients, 26 spinobulbar muscular atrophy (SBMA) patients, 10 spinocerebellar ataxias 2 (SCA2) patients, 61 spinocerebellar ataxias 3 (SCA3) patients, and 50 healthy controls. mtDNA-CN was evaluated by qPCR using primers complementary to the *ND1* gene and the nuclear *β-globin* gene as reference. A significant mtDNA-CN depletion was observed in all poly-Q diseases compared to controls, highlighting a negative correlation between mtDNA-CN and the number of CAG repeats (Table 3). 

Petersen et al. [48] investigated mtDNA-CN in blood leukocytes from a cohort of Danish HD patients. Patients were divided into two groups: sample set 1 to study the alterations in mtDNA-CN between 40 HD mutation carriers versus 28 healthy controls; sample set 2 to investigate the correlation of mtDNA-CN and disease duration in 50 HD patients. mtDNA-CN was evaluated by qPCR using primers to amplify four genes (*Chr7 region*, *ATP synthase*, *ND1*, *ND4*) for sample set 1 and *ATP synthase* and *ND1* genes for sample set 2. In sample set 1, mtDNA-CN was lower in HD carriers compared to healthy controls. Sample set 2 mtDNA-CN showed no significant correlation with disease duration, so authors assessed mtDNA-CN using DNA from 25 HD patients drawn at an earlier point in time. Results showed a biphasic correlation between mtDNA-CN and disease development, with an increase before and a decrease after disease onset (Table 3). 

Jedrak et al. [49] assessed differences between mtDNA-CN in blood leukocytes and in fibroblasts in a Polish cohort of 62 HD patients, 22 presymptomatic HD patients and 79 healthy controls. mtDNA-CN was evaluated by qPCR using primers for mitochondrial 16S rDNA fragments and nuclear *β-globin* genes. Results showed that mtDNA-CN in leukocytes was significantly higher in HD patients compared to controls, while no differences were found between HD patients and presymptomatic HD patients. In fibroblasts, mtDNA-CN was lower in HD patients compared to controls, demonstrating that differences in mtDNA-CN between groups depended on the different examined tissues (Table 3).

### 1.9. Amyotrophic Lateral Sclerosis

Ehinger et al. [50] evaluated mtDNA content and mitochondrial respiration activity in blood samples of 24 ALS patients and 21 healthy controls of Swedish origin. Mitochondrial oxygen consumption was measured in intact and permeabilized cells with Oroboros Instruments (Innsbruck, Austria) while citrate synthase (CS) activity was performed with a commercially available assay kit (CS0720, Sigma–Aldrich, St. Louis, MO, USA). mtDNA-CN was assessed by qPCR using primers directed to the human mitochondrial *COX-1* gene. A progressive decline in mitochondrial electron transport system (ETS) complex I (CI) and complex IV (CIV) was observed in ALS patients with advancing stages of the disease. mtDNA content per cell and CS activity increased in platelets and PBMCs of ALS patients compared to healthy controls, thus hypothesizing that the increase in mitochondrial content could be caused by allostatic compensation for the inadequacy of the mitochondria to comply with cellular energy demands (Table 4). Stoccoro et al. [51] investigated mtDNA-CN and D-loop region methylation in blood specimens of an ALS Italian cohort of *SOD1*, *TARDBP*, *FUS* and *C9orf72* mutation carriers (54 ALS patients, 28 asymptomatic carriers and 32 noncarriers family members). Methylation of the D-loop region was assessed by the methylation-sensitive high-resolution melting (MSHRM) technique, and a sample of completely unmethylated genomic DNA was used as control assay. mtDNA-CN was performed by qPCR, using primers amplifying a nuclear DNA region (hemoglobin subunit β) and a mtDNA region (chrM:3313-3322). mtDNA-CN levels were higher in ALS patients with respect to noncarriers of mutations, while asymptomatic carriers showed an intermediate copy number between the two other groups. D-loop methylation was lower in ALS patients compared to noncarriers of mutations, and asymptomatic carriers showed an intermediate methylation level between ALS patients and noncarrier family members. Furthermore, an inverse correlation between D-loop methylation levels and mtDNA copy number was observed. Moreover, ALS patients with *SOD1* or *C9orf72* mutations showed the highest mtDNA-CN levels (Table 4). 

Harvey et al. [52] evaluated mtDNA-CN levels in 5594 sporadic unrelated ALS patients and 2238 control subjects of different ethnicity. This analysis was performed using whole-genome sequencing (WGS). mtDNA content was estimated using the ratio of reads aligning to mitochondrial DNA: the mtDNA-CN was calculated as double the number of reads aligning to a set of non-repetitive regions throughout the autosome. mtDNA-CN levels were significantly elevated in ALS patients compared to controls, and this value was also correlated with shorter ALS survival. Authors also demonstrated that shorter ALS survival was associated with loss-of-function mutations within the *DNA2* gene, linked to mitochondrial functions. Treating induced pluripotent stem cell (iPSC)-derived motor neurons derived from ALS patients and controls with an inhibitor of DNA2, cellular viability and mitochondrial membrane potential (MMP) were mostly reduced in iPSC-derived motor neurons of controls, thus demonstrating a disease-associated compensatory increase in mtDNA-CN, which is coincident with reduced vulnerability to DNA2 inhibition (Table 4).

### 1.10. Multiple Sclerosis

Al-Kafaji et al. [53] quantified mtDNA-CN in blood samples from 60 Arabian patients with relapsing–remitting MS (RRMS) and 64 healthy controls. qPCR was performed using a primer for the mitochondrial *ND1* gene and for the single-copy nuclear gene *B2M*. mtDNA-CN was, as a result, significantly reduced in RRMS patients compared to controls. Stratification of RRMS patients based on disease duration under or over 10 years revealed that the mtDNA-CN was significantly lower in the group with longer disease duration, indicating that mtDNA-CN may be a potential non-invasive candidate biomarker to investigate mitochondria-mediated neurodegeneration and MS (Table 5).

López-Armas et al. [54] evaluated leukocyte telomere length (LTL) and mtDNA-CN in blood samples of 75 Mexican RRMS patients, 50 with mild expanded disability status scale (SSED) score and 25 with severe SSED score, and 10 were healthy controls. Quantification of mtDNA-CN and measurement of LTL were evaluated by qPCR with the Absolute Human Telomere Length and Mitochondrial DNA Copy Number Dual Quantification qPCR kit (ScienCell Research Laboratories, Carlsbad, CA, USA). Results showed a slight reduction in mtDNA-CN levels and LTL in severe and mild SSED RRMS patients compared to healthy controls. Furthermore, LTL and mtDNA-CN showed a linear correlation in mild–moderate disability RRMS patients (Table 5). 

Sedky et al. [55] measured mtDNA-CN as a biomarker of MS and the progression of the disease in 25 Egyptian RRMS patients and 25 healthy controls. Two peripheral blood samples were collected from each patient, one during the remission phase and the other during the phase of relapse. qPCR was used to amplify the mitochondrial *ND1* gene versus *RNaseP* (nuclear DNA). There was a statistically significant decline in mtDNA-CN during the remission phase as compared to controls. Furthermore, mtDNA-CN levels during the relapse phase were significantly higher than the remission phase, suggesting the ability of mtDNA to differentiate between remission and relapse phases (Table 5).

## 2. Discussion

As we previously said, mtDNA-CN alterations have been associated with many aging processes, suggesting that mtDNA-CN may be a biomarker of mitochondrial health and that mtDNA-CN changes may be associated with aging phenotypes.

In a recent study, Yan et al. utilized Mendelian randomization analysis to explore the causal relationship between TL (a biological process involved in aging) and mtDNA-CN. Their results demonstrated a positive causal link between shortened TL and a decline in mtDNA-CN, providing evidence on how these parameters are involved in cellular senescence and highlighting the dynamic interaction between telomeres and mitochondria in the context of cellular aging [56].

Yang et al. found a significant correlation between mtDNA-CN alterations, metabolic syndrome, and insulin sensitivity. Authors speculated that the loss of mitochondrial function in elderly subjects might lead to lipid accumulation and insulin resistance, corroborating the link between mtDNA-CN changes and aging-related phenotypes [57].

Liu et al. validated the association of blood-derived mtDNA-CN with prevalent and incident cardiovascular disease (CVD) outcomes in eight cohorts of patients of different ethnicities. Authors found a causal association between mtDNA-CN alterations and low-density lipoprotein cholesterol, controlling for coronary heart disease status, indicating that high low-density lipoprotein cholesterol may underlie the complex relationships between mtDNA-CN and vascular atherosclerosis [58].

Although it is not well established how mtDNA defects can influence the onset of neurological diseases, the contribution of mtDNA-CN alteration to the worsening of clinical features detected in these conditions cannot be excluded. 

The main keystone in the landscape of neurodegenerative diseases is the progressive decline in the functionality of neurons, which demand a higher degree of energy, often provided by mitochondria. Therefore, it is reasonable to suppose that the origin and progression of neurological dysfunction can be associated with mitochondrial depletion.

Nowadays, studies about mtDNA-CN can rely on qPCR, which represents a rapid and sensitive technique able to detect mtDNA content. However, the employment of fluids like blood to analyze mtDNA-CN is still debated in the literature because results are often discordant [35,38,41,42,49]. Our purpose was to collect all the main works focusing on this method in order to demonstrate the advantage of mtDNA-CN evaluation in blood to better understand the pathophysiology of neurodegenerative disorders. Several authors investigated fluctuations of mtDNA content in the spectrum of neurodegenerative diseases because this phenomenon could directly influence mitochondrial function and, as a consequence, neurological damage. Many studies demonstrated mtDNA-CN reduction in several brain tissues and the cerebrospinal fluid (CSF) of AD patients compared to controls, showing a correlation between mtDNA-CN, cognitive function and AD pathology [35,59,60,61,62]. Pyle et al. demonstrated that mtDNA-CN content in brain tissues followed the same trend in PBCs isolated from blood of PD patients. In fact, authors found a significant reduction in mtDNA-CN in high-turnover tissues like PBCs of PD patients and in SNp of several tissue necropsies, compared to controls [41]. Results indicated that mtDNA-CN is an important component in the etiology of PD, and its evaluation might be an important early biomarker for PD onset. 

mtDNA-CN has been proved to reflect the cognitive status in subjects affected by MCI. Gorham et al. [39] performed an investigation on a MA population affected by AD and found that mtDNA-CN content was correlated with cognitive impairment, a condition that often affects this ethnicity, considered one of the fastest-aging in the United States. Because MCI represents the stage before the appearance of AD symptoms, it is conceivable that mtDNA-CN analysis could be a useful biomarker for monitoring the cognitive status of patients.

Alteration in mtDNA-CN seemed to be also associated with the *APOE* genotype in AD. In fact, several studies using cellular models, transgenic mouse models and postmortem human brain tissues demonstrated the association between increased oxidative stress and cellular injury in carriers of some *APOE4* alleles [63,64,65]. Gorham et al. reported that mtDNA-CN reduction was slower in AD patients carrying *APOE* ε2/ε3 alleles, supposing that a better preservation in mtDNA-CN levels might be associated with the endowment of this protective *APOE* genotype [39]. 

On the other hand, Liou et al. found a significant correlation, in an AD cohort, between lower mtDNA-CN levels and the presence of *APOE* ε4/ε4, considered a high-risk factor for the onset of that disease [38]. This observation might emphasize the importance of mtDNA-CN evaluation as a way to discriminate oxidative stress status among patients harboring different *APOE* allele genotypes.

In AD, patients carrying different *APOE* genotypes could be more susceptible to oxidative stress, cellular injury and reduced mtDNA-CN [38]. The use of appropriate genetic tests, together with the evaluation of mtDNA-CN levels, might help to better detect the worsening of disease progression.

Accumulation of mutations in mtDNA sites such as D-loop regions and *POLG1* gene frequently predispose PD patients to a drastic decline in mtDNA content, so the examination of these events in detail could be important for the identification of new targets in the treatment of pathology.

TFAM and mtDNA-CN reduction have been observed concurrently in MCI patients, while a decrease in PGC-1α levels has been detected only after AD onset [36]. Since TFAM activity depends on PGC-1α, it is possible that there is a progressive decline of transcription factor cascade over time, and mtDNA-CN depletion could impinge upon this phenomenon.

Mutations in the D-loop region, which controls mtDNA replication and transcription, and in *POLG1*, encoding for mtDNA polymerase required for mtDNA replication and repair, have been associated with mtDNA-CN content in PD [40]. In this study, PD patients containing mutations in D-loop regions and in *POLG1* were more susceptible to a significant mtDNA-CN decrease compared to PD patients without mutations. It is possible that these mutations could have a negative impact on mtDNA content due to the compromising of the OXPHOS pathway and dysfunction of the respiratory chain [40]. For this reason, evaluation of mtDNA-CN could be a useful biomarker to monitor the accumulation of mutations in these mtDNA sites that frequently predispose individuals to age-related diseases. It has been demonstrated that pharmacological therapy can have an impact on mtDNA-CN content, demonstrating that it is possible to modulate mtDNA depletion in neurodegenerative diseases. Liu et al. observed that cholinesterase inhibitors, the standard treatment strategy for AD patients, can have multimodal actions. In fact, mtDNA-CN increased in AD patients receiving cholinesterase inhibitors for more than 3 months versus AD patients with no history of medication [38]. A similar result was observed by Ortega-Vazquez et al. in PD patients. In this study, although mtDNA-CN values in untreated PD patients were similar to controls, an increase in mtDNA content was observed only in PD patients after one or two years of DRT [45]. 

Currently, some effective experimental approaches, mainly in oncology, have been developed with the aim to manipulate mtDNA-CN content for the treatment of several diseases. 

Shen et al. investigated the role of mtDNA-CN alterations in pediatric high-grade gliomas (pHGGs) and developed a potential combination therapy in patient-derived cell cultures and orthotopic xenografts. The synergistic use of dichloroacetate (DCA), a pyruvate dehydrogenase kinase (PDK) inhibitor responsible for regulating mitochondrial activity, together with metformin and radiation therapy, led to a shift in glucose metabolism from glycolysis to OXPHOS, reduced tumor proliferation and improved radiosensitivity in pHGG cells. This study demonstrated that targeting mtDNA-CN may be a novel therapeutic strategy for pHGG treatment [66].

Kubo et al. investigated the role of mtDNA-CN changes in esophageal squamous cell carcinoma (ESCC). Depletion of mtDNA-CN reduced mitochondrial membrane potential (MMP) and increased DNA methylation by upregulation of DNA methyltransferase (DNMT), causing epithelial–mesenchymal transition (EMT) and chemoresistance. The use of a DNMT inhibitor reduced EMT and improved chemotherapy sensitivity in ESCC cell models, suggesting that mechanisms aiming to restore mtDNA-CN content represent a paradigm shift in therapeutic strategies for cancers [67].

Zhuang et al. found that pyrroline-5-carboxylate reductase (PYCR3) regulated mitochondrial gene expression by influencing mtDNA-CN, and its increased expression in triple-negative breast cancer (TNBC) predicted poor prognosis. Selective reduction in mtDNA-CN using the mtDNA replication inhibitor 2′,3′-dideoxycytidine in TNBC cell models resulted in decreased cell proliferation and reverted doxorubicin resistance through apoptosis induction [68].

These data demonstrated that it is possible to restore mitochondrial biogenesis through the evaluation of mtDNA content and drug intervention, suggesting that mtDNA-CN could be a useful biomarker for the monitoring of the therapeutic responses even in patients with neurodegenerative diseases in clinical practice. 

Until now, the evaluation of mtDNA content from blood specimens in poly-Q diseases such as HD gave controversial results [46,47,48,49]. In fact, Chen et al. and Jedrak et al. found increased mtDNA-CN levels in HD patients compared to controls [46,49]. On the other hand, investigations performed by Liu et al. and Petersen et al. demonstrated that mtDNA-CN levels decreased during disease progression [47,48]. Interestingly, Petersen et al. found a biphasic correlation between mtDNA-CN and disease development, with an increase before and a decrease after disease onset [48]. This observation supported the theory proposed by Malik and Czajka, according to which increased mitochondrial biogenesis and replication represent an initial adaptive response to oxidative stress, and mtDNA-CN levels in HD patients could reflect this trend [69]. For this reason, a long-term study of mtDNA-CN content in HD patients could be useful for understanding if the increase in mtDNA content only happens in the first stage of disease or if it persists over time. 

Furthermore, antipsychotics or antidepressants are known to mediate oxidative stress, influencing mitochondrial function and thus representing a confounding factor in term of mtDNA-CN assessment in HD [70]. Mitochondrial function alterations, hence reduced expression of mtDNA encoded genes, have also been detected in ALS, with a decrease proportional to the progression of the disease [71]. In the three studies reported in this review, mtDNA-CN levels were higher in ALS patients compared to controls [50,51,52], corroborating previous evidence of mitochondrial depletion detectable in peripheral blood in such disease [72]. Together with a mtDNA-CN increase, Ehinger et al. also observed a reduction in complex IV activity in ETC, so it is possible that clinical manifestations of neurodegenerative disorders could also reflect a progressive deterioration in mitochondrial machinery [50]. Stoccoro et al. found that a mtDNA-CN increase was also inversely correlated to D-loop methylation levels, strengthening previous evidence that these mechanisms are strictly connected [51]. Furthermore, the increased mtDNA-CN was significantly evident in ALS patients affected by both *SOD1* and *C9orf72* mutations [51], thus demonstrating the importance of investigating and monitoring the pathophysiological changes connected to ALS mutations through mtDNA-CN content analysis.

Harvey et al. identified the nuclear-encoded mitochondrial gene *DNA2*, which is involved in DNA repair and has a large effect on ALS survival [52]. In fact, heterozygous loss-of-function (LoF) changes in this gene could accelerate disease progression, probably due to excessive mtDNA damage. In fact, on treating iPSC-derived motor neuron cells of ALS patients and controls with a DNA2 inhibitor, cellular viability and MMP were reduced only in cell models derived from controls, demonstrating that the effect of DNA2 function on cellular viability is mediated via mitochondrial function in ALS patient-derived iNeurons [52].

The potential contribution of mtDNA-CN measurement, as a hallmark for the worsening of clinical manifestations in ALS, could make this parameter an available biomarker for clinical staging, progression and future therapeutical interventions [52].

ALS patients affected by both *SOD1* and *C9orf72* mutations showed a significant change in mtDNA-CN levels, giving further evidence that impaired mtDNA content might contribute to ALS etiology and that the detection of these mutations could also define the risks and progression of the disease in the other family members [51]. Even in MS, low mtDNA-CN levels represent a signature of high mitochondrial damage and dysfunction [73]. Indeed, mtDNA-CN levels have also the tendency to change on the basis of disease status (relapsing or remitting); this aspect could be crucial to determining the inflammation status in these patients, and the worsening of symptoms can be predicted with more accuracy [55]. In their work, Kafaji et al. demonstrated that monitoring mtDNA-CN content could be useful even in the clinical landscape of disease progression, because this parameter seemed to progressively decrease in disease durations longer than 10 years in RRMS patients [53]. 

In their investigations, Sedky et al. found that when RRMS patients were in remission, mtDNA-CN in peripheral blood decreased significantly compared to a control group. In contrast, mtDNA-CN levels increased during the relapse phase and achieved a statistically significant difference in comparison to mtDNA-CN content during the remission phase [55]. Both these aspects could be extremely informative, because mtDNA-CN evaluation could be a reference parameter to predict and monitor mitochondrial dysfunction and to ameliorate therapeutical interventions especially in neurodegenerative diseases such as MS for which no effective cure is yet available. The different tendencies of mtDNA-CN levels to increase or decrease on the basis of disease status make this value a reliable biomarker in the pathogenicity of MS, helping to detect the resumption of the inflammation process before it happens. 

The typical markers of aging, such as neuroinflammation and neurodegeneration, are also present in MS, and mtDNA-CN analysis, together with LTL measurement in blood specimens, could prove a further element of cognitive function decline in these patients. In fact, Lopez-Armas et al. found a significant linear correlation between LTL shortening and mtDNA-CN decline in their group of RRMS patients affected by mild–moderate disability [54]. For this reason, further studies are needed to streamline the employment of these biomarkers as prognostic factors for MS progression related to cognitive deterioration.

## 3. Conclusions

The aim of our review is to collect all recent studies that investigate the usefulness of mtDNA-CN as a biomarker in the pathophysiological mechanisms of neurodegenerative diseases.

As in aging, one of the main signatures for the onset of neurodegeneration is represented by mitochondrial dysfunction and depletion, so it is reasonable to suppose that alteration in mtDNA-CN levels might represent a signal of onset and progression of these disorders that can be easily detected in blood specimens.

The detection of mtDNA-CN in blood, as well as in brain tissues and CSF, could represent a useful approach to monitor pathological status in patients at risk to develop neurodegenerative diseases, and it could give the opportunity to exploit this information for a proper and well-timed therapeutic intervention.

## 4. Future Directions

All this evidence suggests the importance of exploring the complex link existing between mtDNA-CN changes and neurodegenerative disorders. We believe that understanding the mitochondrial contributions to neurodegeneration could offer the opportunity to increase our knowledge of the complex mechanisms of neuronal degenerations, translating this information into new diagnostic tools and future therapeutic interventions.

## Figures and Tables

**Table 1:** mtDNA-CN evaluation in AD disease. Main findings of studies assessing the impact of mtDNA-CN evaluation in blood samples of AD patients.

References	Disease	Ethnicity	Samples	Aim	Methods	Main Findings	Conclusions
Rodriguez-Santiago et al., 2001 [35]	AD	Spanish	Blood samples of 17 AD patients and 11 healthy controls	Evaluation of mtDNA-CN to investigate mtDNA content in AD patients	qPCR was used to quantify mtDNA-CN vs. nuclear ribosomal *18S* gene	mtDNA-CN was similar in AD patients and controls	The contribution of mtDNA defects to the pathophysiology of AD remains elusive
Delbarba et al., 2016 [36]	AD	Spanish/Italian	Blood samples of 20 AD patients, 24 MCI patients, 30 healthy controls DNA samples of 276 AD patients, 70 MCI patients, 248 healthy controls	Evaluation of mtDNA-CN to detect differences between AD, MCI and healthy controls	mtDNA-CN was evaluated by qPCR as the amount of mitochondrial *Cytochrome B* gene normalized to nuclear *36B4* gene	mtDNA-CN decreased in MCI and in AD patients of both groups compared to healthy controls	Low mtDNA-CN was correlated with cognitive decline. This change could represent a feature of early events occurring in AD
Lunnon et al., 2017 [37]	AD	English	Blood samples of 28 AD patients, 31 MCI patients and 28 healthy controls	Evaluation of mtDNA-CN to establish if mtDNA content was altered in AD	mtDNA-CN was measured by qPCR using primers complementary to unique regions of mitochondrial genome. mtDNA content was quantified as the ratio of mitochondrial genome to nuclear genome	No difference in mtDNA-CN was found between AD, MCI and healthy controls	There was no change in steady-state numbers of mitochondria in spite of an increased expression of some mitochondrial-encoded OXPHOS genes
Liou et al., 2021 [38]	AD	Taiwanese	Blood samples of 600 AD patients and 601 healthy controls carrying different allele genotypes for *APOE4* gene (no E4 allele; heterozygous E4 allele; homozygous E4 allele)	Evaluation of mtDNA-CN as biomarker to predict AD onset	mtDNA content was measured by qPCR using primers complementary to the sequence of *ND1* gene and normalized to *β-actin* gene	mtDNA-CN decreased in AD group compared to controls. Low mtDNA-CN was correlated with homozygous E4 allele in AD cohort	There was an association between AD, the presence of *APOE4* allele and mtDNA-CN content
Gorham et al., 2024 [39]	AD	NHW MA	Blood samples (Buffy Coat) for both groups divided as: NHW (63AD, 28 MCI, 129 NC) MA (31 AD, 72 MCI, 149 NC)	Evaluation of mtDNA-CN to identify population-specific differences as indicators of mitochondrial dysfunction associated with AD	mtDNA content was measured by qPCR via the MinArc and nuclear *B2M* gene and quantified as the ratio of mitochondrial genome to nuclear genome	mtDNA-CN decreased in AD patients compared to NC in MA cohort; mtDNA-CN increased in MCI compared to NC in NHW cohort	Increased mitochondrial dysfunction, as indicated by decreased mtDNA-CN, may play a direct role in cognitive decline in MA cohort. In NHW cohort, increased mtDNA-CN is associated with progressive cognitive decline

AD: Alzheimer’s Disease; mtDNA-CN: Mitochondrial DNA Copy Number; qPCR: Quantitative Real Time PCR; vs.: versus; MCI: Mild Cognitive Impairment; OXPHOS: Oxidative Phosphorylation; NHW: non-Hispanic White; MA: Mexican American; NC: Cognitively Normal; MinAr.

**Table 2:** mtDNA-CN evaluation in PD disease. Main findings of studies assessing the impact of mtDNA-CN evaluation in blood samples of PD patients.

References	Disease	Ethnicity	Samples	Aim	Methods	Main Findings	Conclusions
Gui et al., 2015 [40]	PD	Chinese	Blood samples of 414 PD patients and 231 healthy controls	Evaluation of mtDNA-CN to understand whether alteration in mtDNA content occurs along with mutations in D-loop region in PD	mtDNA-CN was evaluated by qPCR using primers complementary to D-loop region and normalized to *β-actin* gene	mtDNA-CN decreased in PD group compared to controls Lower mtDNA-CN levels were detected in older PD patients mtDNA-CN was lower in PD patients mutated for poly C stretch or heavy-strand replication origin site	Significant association between lower mtDNA-CN and PD indicated that decreased mtDNA content may be involved in PD and could have potential prognostic significance in PD progression
Pyle et al., 2016 [41]	PD	Caucasian	Blood samples of 363 PD patients and 262 healthy controls	Evaluation of mtDNA-CN to establish if there was an alteration of mitochondrial content in PD	mtDNA-CN was evaluated by multiplex qPCR of the mitochondrial genes *MTND1*, *MTND4* and nuclear *B2M* gene	mtDNA-CN decreased in PD group compared to controls	Mitochondrial biogenesis, evaluated as mtDNA-CN, may be a viable diagnostic predictor of PD
Stoccoro et al., 2021 [42]	PD	Italian	Blood samples of 30 PD patients and 30 healthy controls	Evaluation of mtDNA-CN to address the potential involvement of mtDNA changes in PD	mtDNA-CN was evaluated by qPCR using primers specific for nuclear and mitochondrial regions	mtDNA-CN did not differ between PD group compared to controls	Further studies are needed to shed light on the potential mtDNA-CN change in PD
Muller-Nedebock et al., 2022 [43]	PD	African	Blood samples of 72 PD patients and 79 healthy controls	Evaluation of mtDNA-CN alteration in African-ancestry PD patients	ddPCR was performed to quantify mtDNA-CN levels as a ratio of mitochondrial gene (*MT-TL1*) vs. nuclear gene (*B2M*)	mtDNA-CN increased in PD group compared to controls	Differences observed in mtDNA-CN between the present and past studies may be a result of unaccounted-for factors and variability in study designs
Asghar et al., 2022 [44]	PD	Sweden	Blood samples of 100 PD patients and 12 healthy controls	Evaluation of mtDNA-CN to investigate the association between PD and mitochondrial alterations	mtDNA-CN was evaluated by qPCR using Relative Human mitochondrail DNA copy number quantification kit (ScienceCell, Carlsbad, CA, USA)	mtDNA-CN decreased in PD group compared to controls	Mitochondrial dysfunction, detected as mtDNA-CN in blood, may be a valuable marker to assess the risk of PD onset
Ortega-Vazquez et al., 2023 [45]	PD	Mexican	Blood samples of 27 PD patients and 22 healthy controls	Case-control study for mtDNA-CN evaluation in PD patients before and after dopaminergic treatment	mtDNA-CN was evaluated by qPCR using primers complementary to *ND3* gene and nuclear *TH* gene as reference	mtDNA-CN in untreated PD patients was similar to controls mtDNA-CN increased in PD patients after dopaminergic treatment	mtDNA-CN may contribute to the pathophysiology of PD with potential opportunities for future therapeuthic interventions

PD: Parkinson Disease.

**Table 3:** mtDNA-CN evaluation in HD. Main findings of studies assessing the impact of mtDNA-CN evaluation in blood samples of HD patients.

References	Disease	Ethnicity	Samples	Aim	Methods	Main Findings	Conclusions
Chen et al., 2007 [46]	HD	Taiwanese	Blood samples for leukocyte isolation of 16 HD patients and 36 healthy controls	Evaluation of mtDNA content to investigate if pathological changes in HD may also be present in peripheral tissues	mtDNA-CN was evaluated by qPCR using primers to amplify total mtDNA and nuclear *GAPDH* gene	mtDNA-CN resulted, increased in HD patients compared to controls	High mtDNA-CN may be a compensatory response to increased oxidative stress, and treatment aimed at restoring mitochondrial function may be beneficial for HD patients
Liu et al., 2008 [47]	HD	Taiwanese	Blood samples for leukocyte isolation of 17 HD patients and 50 healthy controls	Evaluation of mtDNA content as a biomarker to predict the severity of clinical manifestation in poly-Q diseases	mtDNA-CN was evaluated by qPCR using primers complementary to *ND1* gene and nuclear *β-globin* gene as reference	mtDNA-CN resulted, decreased in HD patients compared to controls	mtDNA content correlated with the lenght of GAG repeat and may serve as an index for the severity of poly-Q disorders
Petersen et al., 2014 [48]	HD	Danish	Blood samples for leukocyte isolation of 50 HD patients 41 HD mutations carriers 28 healthy controls	Evaluation of mtDNA-CN to investigate: mtDNA-CN in HD carriers vs. healthy controls; mtDNA-CN vs disease duration in HD patients	mtDNA-CN was evaluated by qPCR using primers to amplify Chr7 region, *ATP synthase*, *ND1* and *ND4* genes	mtDNA-CN was lower in HD carriers compared to healthy controls mtDNA-CN in HD patients decreased during the course of the disease	mtDNA-CN might be a biomarker of HD disease progression but there is a need of more samples taken over a longer period, preferably before disease onset
Jedrak et al., 2017 [49]	HD	Polish	Blood samples for leukocyte isolation of 62 HD patients, 22 presymptomatic HD patients and 79 healthy controls	Evaluation of mtDNA-CN to investigate if there are differences in mtDNA levels in HD patients	mtDNA-CN was evaluated by qPCR using primers for mitochondrial 16S rDNA fragment and nuclear *β2M* gene	mtDNA-CN progressively increased from presymptomatic to symtomatic HD patients compared to controls	mtDNA-CN content has to be better investigated using larger sample sizes, and the type of tissue used for mtDNA isolation could significantly affect results of mtDNA-CN in HD

HD: Huntington Disease.

**Table 4:** mtDNA-CN evaluation in ALS. Main findings of studies assessing the impact of mtDNA-CN evaluation in blood samples of ALS patients.

References	Disease	Ethnicity	Samples	Aim	Methods	Main Findings	Conclusions
Ehinger et al., 2015 [50]	ALS	Swedish	Blood samples of 24 ALS patients and 21 healthy controls	Evaluation of complex IV activity in ALS patients	mtDNA-CN was measured by SYBR Green PCR using primers for *COX1* gene	mtDNA-CN slightly increased in ALS patients compared to controls	In ALS blood cells, the study of mitochondrial function could be a future biomarker for the disease
Stoccoro et al., 2018 [51]	ALS	Italian	Blood samples of 54 ALS patients, 28 asymtomatic carriers and 32 noncarrier family members	Evaluation of mtDNA-CN to investigate mtDNA content in ALS patients	qPCR was performed using primers complementary to mtDNA region (chrM:3313-3322) and nuclear region (hemoglobin subunit β)	mtDNA-CN was higher in ALS patients compared to noncarrier family member; asymptomatic carriers showed intermediate mtDNA-CN level compared to other groups.	There was an inverse correlation between mtDNA-CN levels and D-loop methylation levels
Harvey et al., 2024 [52]	ALS	English, Belgian, German, Irish, Spanish, Italian, Turkish, American, Dutch	Blood samples of 5594 sporadic ALS patients and 2238 healthy controls	Evaluation of mtDNA-CN in ALS patients	WGS was used for measure mtDNA-CN levels	mtDNA-CN was higher in ALS patients compared to controls.	Mitochondrial function was a therapeutic target for amelioration of disease severity

ALS: Amyotrophic Lateral Sclerosis.

**Table 5:** mtDNA-CN evaluation in MS. Main findings of studies assessing the impact of mtDNA-CN evaluation in blood samples of MS patients.

References	Disease	Ethnicity	Samples	Aim	Methods	Main Findings	Conclusions
Al-Kafaji et al., 2020 [53]	MS	Arabian	Blood samples of 60 RRMS patients and 64 healthy controls	mtDNA-CN evaluation in RRMS patients to determine if mtDNA content is associated with disease duration	mtDNA-CN was measured by qPCR as the amount of mitochondrial *ND1* gene normalized to nuclear *B2M* gene	mtDNA-CN was reduced in RRMS patients compared to controls; decreased mtDNA-CN was observed in patients with long-term disease duration	mtDNA-CN reduction in RRMS patients was a direct consequence of impaired mitochondrial function, and this event is important in MS pathogenicity and progression
Del Carmen Lòpez-Armas et al., 2023 [54]	MS	Mexican	Blood samples of 75 RRMS patients (50 with mild SSED and 25 with severe SSED) and 10 healthy controls	mtDNA-CN assessment as potential biomarkers in RRMS patients	mtDNA-CN was evaluated by qPCR using ScienceCell kit	There was a slight reduction in mtDNA-CN levels in severe and mild SSED RRMS patients compared to healthy controls	Authors suggested to use a larger sample size to establish the clinical utility of mtDNA-CN assessment in MS
Sedky et al., 2023 [55]	MS	Egyptian	Blood samples of 25 RRMS patients and 25 healthy controls	Evaluation of mtDNA-CN as a potential biomarker of MS onset and progression	qRT-PCR was used to amplify mitochondrial *ND1* gene vs. nuclear *RNaseP* gene	In RRMS patients in remission, mtDNA-CN decreased compared to control group. In RRMS patients in relapse, mtDNA-CN increased compared to controls	mtDNA-CN reduction in peripheral blood from RRMS patients might be a significant event in the overview of MS.

MS: Multiple Sclerosis.

## List of Abbreviations

CNS	Central nervous system.
mtDNA-CN	Mitochondrial DNA copy number.
AD	Alzheimer’s disease.
PD	Parkinson’s disease.
HD	Huntington’s disease.
ALS	Amyotrophic lateral sclerosis.
MS	Multiple sclerosis.
Aβ	Amyloid-β.
TDP43	TAR DNA-binding protein 43.
mtDNA	Mitochondrial DNA.
OXPHOS	Oxidative phosphorylation.
D-loop	Displacement loop.
OriH	Origin of replication.
ATP	Adenosine triphosphate.
ROS	Reactive oxygen species.
ER	Endoplasmic reticulum.
SASPs	Senescence-associated secretory phenotypes.
mPTPs	Mitochondrial permeability transition pores.
NLRP3	Nod-like receptor pyrin domain 3.
NGS	Next-generation sequencing.
BER	Base excision repair.
ETC	Electron transport chain.
Htt	Huntingtin.
qPCR	Quantitative real-time PCR.
PBMCs	Peripheral blood mononuclear cells.
MCI	Mild cognitive impairment.
CYT B	Cytochrome B.
CYT C	Cytochrome C.
MMSE	Mini-mental state examination.
PGC-1α	Peroxisome proliferator-activated receptor gamma coactivator 1-alpha.
TFAM	Mitochondrial transcription factor A.
B2M	Beta 2 microglobin.
TBARS	Thiobarbituric acid reactive substances.
MA	Mexican–American.
NHW	Non-Hispanic white.
MinArc	Mitochondrial minor arc.
POLG1	DNA polymerase subunit gamma 1.
SNpc	Substantia nigra pars compacta.
Fc	Frontal cortex.
PBCs	Peripheral white blood cells.
MS-HRM	Methylation-sensitive high-resolution melting technique.
ddPCR	Droplet digital polymerase chain reaction.
TL	Telomere length.
PDD	Parkinson’s disease dementia.
DLB	Dementia with Lewy bodies.
LEDD	Levodopa equivalent daily dose.
DRT	Dopaminergic replacement therapy.
TH	Tyrosine hydroxylase.
8-OHdG	8-hydroxydeoxyguanosine.
MDA	Plasma malondialdehyde.
GPx	Glutathione peroxidase.
HPLC	High-performance liquid chromatography.
HPLC-ECD	High-performance liquid chromatography–electrochemical detector.
SBMA	Spinobulbar muscular atrophy.
SCA2	Spinocerebellar ataxias 2.
SCA3	Spinocerebellar ataxias 3.
CS	Citrate synthase.
ETS	Electron transport system.
CI	Complex I.
CIV	Complex IV.
MSHRM	Methylation-sensitive high-resolution melting.
WGS	Whole-genome sequencing.
iPSC	Induced pluripotent stem cells.
MMP	Mitochondrial membrane potential.
RRMS	Relapsing–remitting multiple sclerosis.
LTL	Leukocyte telomere length.
SSED	Expanded disability status scale.
CVD	Cardiovascular disease.
pHGG	Pediatric high-grade glioma.
DCA	Dichloroacetate.
PDK	Pyruvate dehydrogenase kinase.
ESCC	Esophageal squamous cell carcinoma.
MMP	Mitochondrial membrane potential.
DNMT	DNA methyltransferase.
EMT	Epithelial–mesenchymal transition.
PYCR3	Pyrroline-5-carboxylate reductase.
TNBC	Triple-negative breast cancer.
CSF	Cerebrospinal fluid.
DNA2	DNA replication helicase/nuclease 2.
LoF	Loss of function.
